# Transcriptome Sequencing and *De Novo* Analysis for Yesso Scallop (*Patinopecten yessoensis*) Using 454 GS FLX

**DOI:** 10.1371/journal.pone.0021560

**Published:** 2011-06-24

**Authors:** Rui Hou, Zhenmin Bao, Shan Wang, Hailin Su, Yan Li, Huixia Du, Jingjie Hu, Shi Wang, Xiaoli Hu

**Affiliations:** Key Laboratory of Marine Genetics and Breeding (MGB), Ministry of Education, College of Marine Life Sciences. Ocean University of China, Qingdao, China; Biodiversity Insitute of Ontario-University of Guelph, Canada

## Abstract

**Background:**

Bivalves comprise 30,000 extant species, constituting the second largest group of mollusks. However, limited genetic research has focused on this group of animals so far, which is, in part, due to the lack of genomic resources. The advent of high-throughput sequencing technologies enables generation of genomic resources in a short time and at a minimal cost, and therefore provides a turning point for bivalve research. In the present study, we performed *de novo* transcriptome sequencing to first produce a comprehensive expressed sequence tag (EST) dataset for the Yesso scallop (*Patinopecten yessoensis*).

**Results:**

In a single 454 sequencing run, 805,330 reads were produced and then assembled into 32,590 contigs, with about six-fold sequencing coverage. A total of 25,237 unique protein-coding genes were identified from a variety of developmental stages and adult tissues based on sequence similarities with known proteins. As determined by GO annotation and KEGG pathway mapping, functional annotation of the unigenes recovered diverse biological functions and processes. Transcripts putatively involved in growth, reproduction and stress/immune-response were identified. More than 49,000 single nucleotide polymorphisms (SNPs) and 2,700 simple sequence repeats (SSRs) were also detected.

**Conclusion:**

Our data provide the most comprehensive transcriptomic resource currently available for *P. yessoensis*. Candidate genes potentially involved in growth, reproduction, and stress/immunity-response were identified, and are worthy of further investigation. A large number of SNPs and SSRs were also identified and ready for marker development. This resource should lay an important foundation for future genetic or genomic studies on this species.

## Introduction

Lophotrochozoa are a major group of protostome animals, and Mollusca are the largest phylum of this clade. Bivalves may have appeared as early as the Cambrian period [1]. They comprise 30,000 extant species, constituting the second largest group of mollusks [2]. In spite of their species abundance and diverse geographical distribution, limited research has been conducted on this particular group of animals. To date, many of bivalve studies have been limited to a few well-studied species. Genetic or genomic studies on a broader range of bivalve species would clearly enable a better understanding of the phylogeny, speciation and diversification of bivalves. Fortunately, the recent advent of high-throughput sequencing technologies, which can dramatically speed up genetic and genomic studies on potentially any organisms, provides a turning point for bivalve research.

The Yesso scallop, *Patinopecten yessoensis* (Jay, 1857), is a cold water bivalve and naturally distributes along the coastline of northern Japan, the Far East of Russian and the northern Korean Peninsula. It is the main scallop species cultured in Japan and has become one of the most important maricultural shellfish in the north of China since it was introduced in 1982 [3]. Preliminary genetic studies on *P. yessoensis* have recently been performed, which focused on development of genetic markers [4], [5], construction of genetic maps [6], [7], and characterization of functional genes [8], [9]. As an economically important aquacultural species, understanding of genetic mechanisms involved in the growth, reproduction and immunity of *P. yessoensis* is currently active research areas. However, these research areas have long suffered from one of the challenges of systematic biology studies, namely, the lack of genomic resources such as genome or transcriptome sequences.

The genome size of *P. yessoensis* is ∼1.7 Gb [10]. Sequencing of such large genome remains expensive even using next-generation sequencing technologies. Expressed sequence tag (EST) sequencing represents an attractive alternative to whole-genome sequencing because EST sequencing only analyzes transcribed portions of the genome, while avoiding non-coding and repetitive sequences that can make up much of the genome. In addition, EST sequencing is also an effective way to develop ‘functional’ genetic markers that are very useful for genetic or genomic studies. There are ∼7,600 EST sequences available for *P. yessoensis* in the GenBank database, but a comprehensive description of its transcriptome remains unavailable. The increased throughput of next-generation sequencing technologies, such as the massively parallel 454 pyrosequencing, allows increased sequencing depth and coverage, while reducing the time, labor, and cost required [11]–[13]. These technologies have shown great potential for expanding sequence databases of not only model species [14]–[18] but also non-model organisms [19]–[24].

In the present study, we performed *de novo* transcriptome sequencing for *P. yessoensis* using the 454 GS FLX platform. Approximately 25,000 different transcripts and a large number of SSRs and SNPs were identified. Our EST database should represent an invaluable resource for future genetic and genomic studies on this species.

## Results and Discussion

### Sequence analysis and assembly

**Figure 1:**
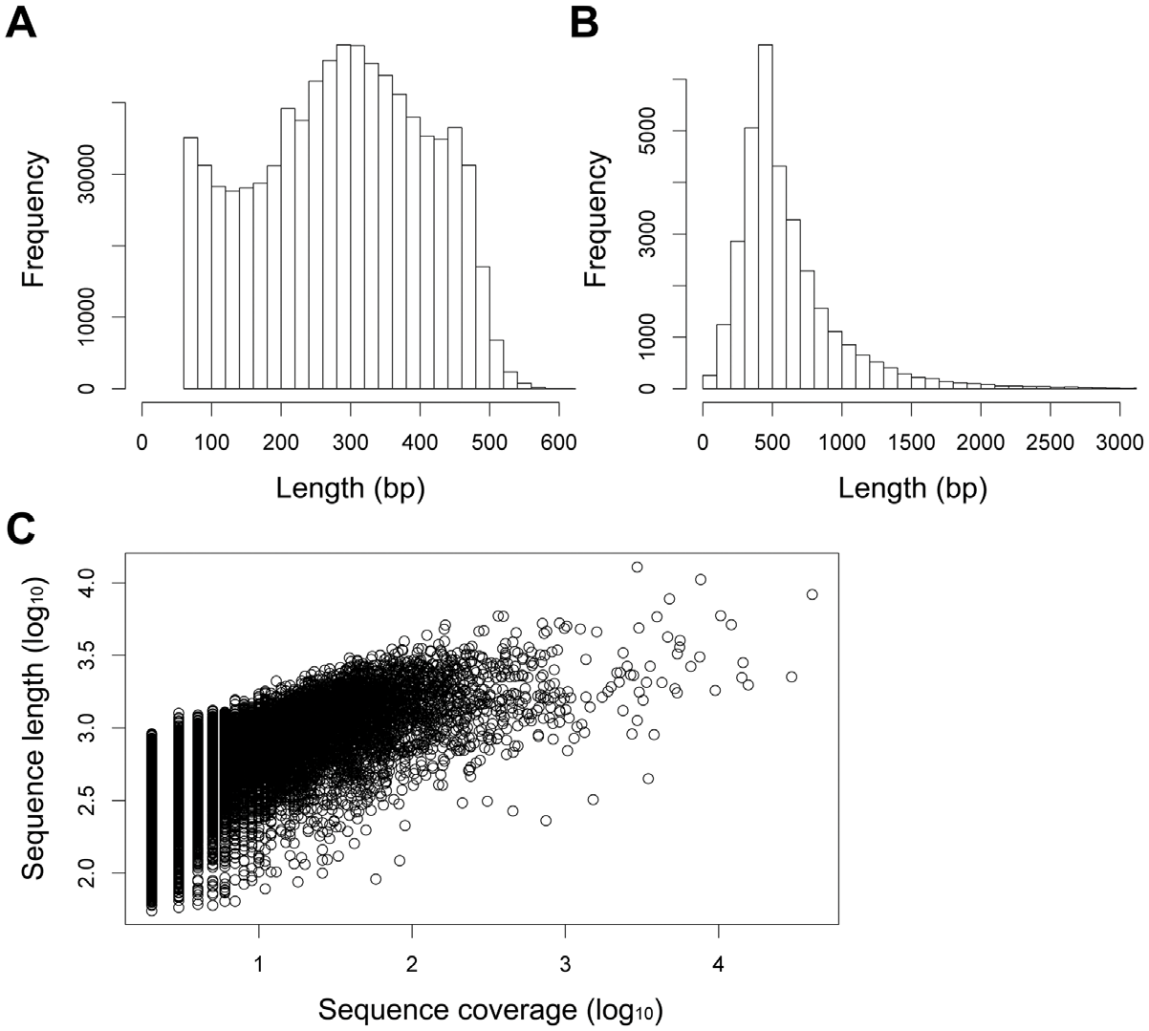
Overview of the *P. yessoensis* transcriptome sequencing and assembly. (A) Size distribution of 454 sequencing reads after removal of adaptor and short sequences (<60 bases). (B) Size distribution of contigs. (C) Log-log plot showing the dependence of contig lengths on the number of reads assembled into each contig.

A mixed cDNA sample representing diverse developmental stages and adult tissues of *P. yessoensis* was prepared and sequenced using the 454 GS FLX platform for a single sequencing run. This sequencing run produced 970,422 (∼304 Mb) raw reads with an average length of 313 bases. An overview of the sequencing and assembly process is presented in Table 1. After removal of adaptor sequences, 882,588 (∼234 Mb) reads remained with an average length of 265 bases. The removal of short reads (<60 bases) reduced the total number of reads to 805,330 (∼231 Mb); the average read length was 287 bases. The cleaned reads produced in this study have been deposited in the NCBI SRA database (accession number: SRA027310). These results revealed that 83.0% of raw reads contained useful sequence data. The size distribution for these trimmed, size-selected reads is shown in Fig. 1A. Overall, 90.4% (728,265) of the clean reads were between 100 and 500 bp in length.

**Table 1:** Summary of 454 transcriptome sequencing and assembly for *P. yessoensis.*

	Reads (n)	Bases (Mb)	Average length (bp)
Raw sequencing reads	970,422	303.8	313.1
Clean reads	805,330	230.7	286.5
Contigs	32,590	20.2	618.4
Singletons	106,807	28.1	262.9
Total	139,397	48.3	346.0

Assembly of the 805,330 clean reads produced 32,590 contigs, ranging from 60 to 12,879 bp in size, with an average size of 618 bp. These contigs incorporated 87% of high-quality reads, which resembled the assembly efficiency of 454 reads (80–92%) reported in previous studies [21], [22], [25]–[27]. The size distribution of these contigs is shown in Fig. 1B. Among these contigs, 16,569 (50.8%) were longer than 500 bp, of which 3,977 (12.2%) were longer than 1,000 bp. These results demonstrated the effectiveness of 454 pyrosequencing in rapidly capturing a large portion of the *P. yessoensis* transcriptome. The sequencing depth was 5.8 X on average. As expected for a randomly fragmented transcriptome, there was a positive relationship between the length of a given contig and the number of reads assembled into it (Fig. 1C). The remaining 106,807 high-quality reads were retained as singletons.

About 7.7% of the reads produced in this study matched to microbes, and over 83% of the microbial transcripts were turned out to come from the embryo and larval library, of which samples were collected directly from non-sterile seawater. It seems very plausible that the majority of identified microbial sequences were caused by microbial contamination from seawater. Therefore, these microbial sequences have been removed from the procedures of functional annotation, and SSR and SNP mining.

### Sequence annotation

We utilized several complementary approaches to annotate the assembled sequences. First, the assembled sequences were compared against the public Nr and Swiss-Prot databases using BlastX (E-value<1e-4). Of the 139,397 assembled sequences, 38,942 (14,638 contigs plus 24,304 singletons) had a significant matches (Table S1) corresponding to 25,237 unique accession numbers, of which 6,622 were matched by multiple queries without overlap. These 6,622 subject sequences were matched by 20,327 different query sequences (3.1 matched queries per subject, on average). Additionally, 24,304 singletons showed significant matches to 17,204 unique accession numbers, of which 13,661 (79.4%) were not found among contigs, suggesting that most of singletons contained useful gene information which could not be obtained from contigs. It could be due to the fact that many genes in the transcriptome are expressed at levels low enough to hinder adequate sampling for 454 sequencing.

The percentage of sequences without annotation information in this study was considerable (approximately 72.1%). The poor annotation efficiency could be due to the insufficient sequences in public databases for phylogenetically closely related species to date. For example, 461 (1.2%) hits were matched to *P. yessoensis*; 228 (0.6%) to *C. farreri* (Zhikong scallop); 182 (0.5%) to *C. gigas* (Pacific oyster); and 176 (0.5%) to *A. irradians* (Bay scallop). Only 4.1% of the BLAST hits matched to Bivalvia class in total. On the other hand, because the significance of the BLAST comparison depends in part on the length of the query sequence, short reads obtained from sequencing would rarely be matched to known genes [13]. In this study, almost half of the assembled sequences were not very long (48.3%<300 bp), which might be too short to allow for statistically meaningful matches. For sequences longer than 300 bp, annotation rate was 39.0%, while for sequences longer than 1 kb, the proportion increased to 67.6% (Table 2). Additionally, sequences without annotations may represent poorly conserved regions (e.g., un-translated regions (UTRs)) in *P. yessoensis*.

**Table 2:** Functional annotation of the *P. yessoensis* transcriptome.

	ESTs (unique genes)		
	All sequences	≥300 bp	≥1000 bp
Total number of sequences	139,397	72,077	3,978
ESTs with BLAST matches against Nr	38,536 (28,864)	27,971 (22,067)	2,783 (2,549)
ESTs with BLAST matches against Swiss-Prot	29,195 (17,647)	21,932 (14,472)	2,455 (2,259)
ESTs assigned with GO terms	15,530(9,290)	17,027 (7,921)	1,873 (1,607)
ESTs assigned with EC numbers	4,846 (3,209)	4,454 (3,078)	990 (894)

**Figure 2:**
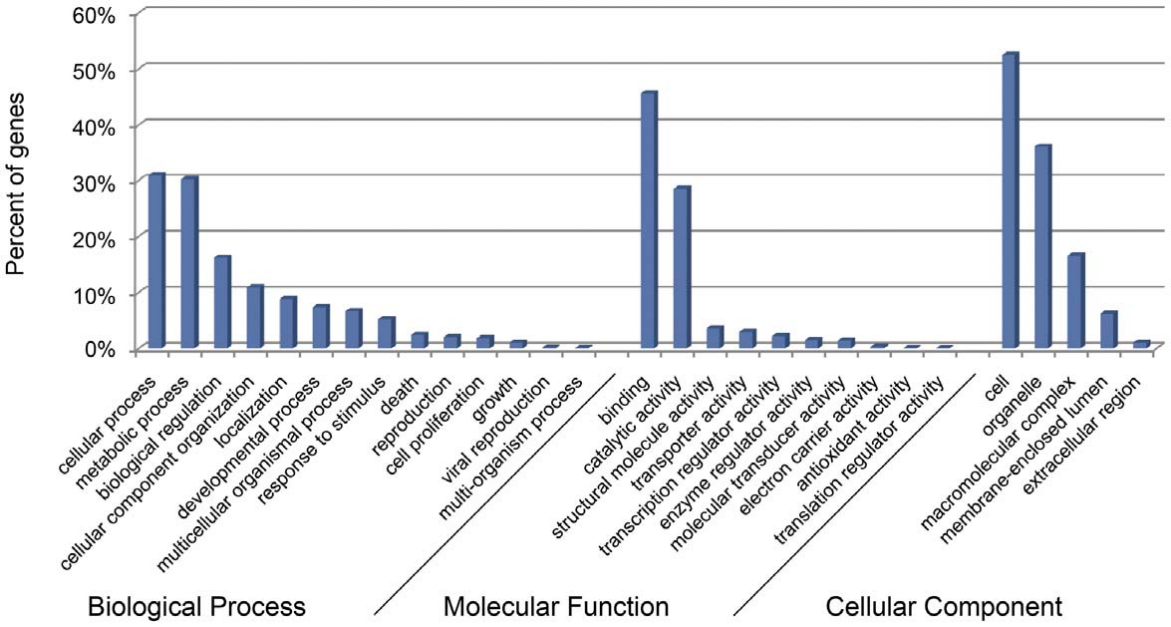
Functional annotation of assembled sequences based on gene ontology (GO) categorization. GO analysis was performed at the level 2 for three main categories (cellular component, molecular function and biological process).

Secondly, Gene Ontology (GO) [28] analysis was carried out, which provides a dynamic, controlled vocabulary and hierarchical relationships for the representation of information on molecular function, cellular component and biological process, allowing a coherent annotation of gene products. Of 21,414 annotated sequences in Swiss-Prot, 15,530 (72.5%) were assigned with one or more GO terms. In total, 81,121 GO assignments were finally obtained, with 37.1% for biological processes, 32.4% for molecular functions, and 30.4% for cellular components. For biological processes, genes involved in cellular process (GO: 0009987) and metabolic process (GO: 0008152) were highly represented. For molecular functions, binding (GO: 0005488) were the most represented GO term, followed by catalytic activity (GO: 0003824). Regarding cellular component, the most represented categories were cells (GO: 0005623) and organelles (GO: 0043226) (Fig. 2).

Besides GO analysis, KEGG [29] pathway mapping based on enzyme commission (EC) numbers for assignments was also carried out for the assembled sequences, which is an alternative approach to categorize genes functions with the emphasis on biochemical pathways. EC numbers were assigned to 4,846 unique sequences, which were involved in 244 different pathways. Summary of the sequences involved in these pathways was included in Table S2. Of these 4,846 sequences with KEGG annotation, 45.5% were classified into the genetic information processing (GIP), with most of them involved in replication and repair, folding, sorting and degradation, transcription, and translation. Sequences classified into the metabolism accounted for 42.8% of the KEGG annotated sequences. The well-represented metabolic pathways were enzyme families, carbohydrate metabolism, amino acid metabolism, and energy metabolism. Cellular processes were represented by 18.3% of the KEGG annotated sequences. The cell motility, cell growth and death, immune system, and endocrine system were well represented. Additionally, 15.2% of the sequences were classified into environmental information processing (EIP) including signal transduction, signaling and interaction molecules, and membrane transport.

### Functional genes involved in growth, reproduction, stress and immunity

For many aquaculture animals like scallop, economic traits like growth and reproduction are of particular interest to the researchers. The sequence and annotation information from BLAST, GO and KEGG annotations all provided valuable gene sources for the study of molecular basis that underline these economic traits of *P. yessoensis*. Transcripts putatively take part in growth (GO: 0040007) and reproduction (GO: 0000003) were found in our 454 database (Table S3). Among them, genes encoding different groups of growth factors and their receptors involved in cell growth were identified, such as epidermal growth factor domains and receptors, transforming growth factors and receptors, insulin-like growth factor receptors and fibroblast growth factor and receptors. Interestingly, several transcripts encoding for MAP kinase signal-integrating kinase 1 (Mnk1) were also identified. This gene is a transcriptional and translational regulator, and is an important modulator of cell growth and proliferation [30].

Regarding reproduction, genes encoding DEAD-box family members such as vasa , PL10 and eIF4A that function in germ cell development and reproductive regulation, and Piwi-like proteins that are responsible for maintaining the stability of cells division rates in germ cells were identified. Several transcripts involved in gonad development were also observed. For example, vitellogenin (vtg), the precursor of egg yolk proteins that are sources of nutrients during embryonic development [31], was highly expressed in the female gonad. Interestingly, vtg expression was also detected at a very low level in the other three tissues including male gonad. In the male gonad, a transcript homologous to the sperm-specific H1/protamine-like protein was highly expressed. This protein is responsible for compacting sperm DNA into a highly condensed, stable, inactive complex and is involved in chromatin remodeling and/or transcriptional regulation during spermiogenesis [32]. This transcript was also expressed at a very low level in other tissues, but absent in female gonad.

The identification of a number of stress and immune-related transcripts (Table S3) are also of interest to scallop researchers because of increasing environmental pressures on scallop populations resulting from the increasing use of coastal zones and from the devastating effects of diseases. Both KEGG and GO analysis identified transcripts potentially involved in responses to environmental pressure and stimulus. The GO annotation identifed 812 sequences that are potentially related to stimulus responses (GO: 0050896). The Hsp families playing an important role in thermal tolerance [33] were the most abundant transcripts in this category. They are necessary for protein folding, multimer dissociation and association, translocation of proteins across membranes, and regulation of the heat shock response [34], [35]. Since the *P. yessoensis* is a cold water species, high expression of Hsps could possibly promote more efficient folding of proteins at low temperatures [36], [37]. The stress-associated endoplasmic reticulum protein (SERP2) is also highly expressed and this gene may be linked to antioxidant capacity, and stabilisation of membranes in response to stress [24]. For KEGG analysis, 181 sequences were classified into immune system, and they were involved in 14 immune-response pathways.

Overall, functional analysis of our 454 database identified candidate genes potentially involved in growth, reproduction, stress and immunity. Further experiments are needed to validate the functions and expression patterns of these candidate genes, and investigate their potential roles in the gonad development and reproduction.

### SSR and SNP discovery

As an important aquacultural shellfish in China, the application of marker-assisted selection (MAS) or genome-wide marker-assisted selection (G-MAS) in the *P. yessoensis* breeding program is expected to be a fertile research area. However, few genetic markers are currently available for this species [4], [5]. The transcriptome data obtained by 454 sequencing provided an excellent source for mining and development of gene-associated markers. [13], [32], [38], [39].

In total, 2,748 SSRs were identified from the assembled sequences (Table 3). Of 2,494 SSR-containing sequences, 420 (16.8%), had been annotated, and can be considered as priority candidates for maker development. The most frequent repeat motifs were trinucleotides, which accounted for 39.4% of all SSRs, followed by dinucleotides (21.1%), tetranucleotides (15.5%), pentanucleotides (14.6%), and hexanucleotides (9.4%). Based on the distribution of SSR motifs, AT motifs represented the most abundant dinucleotide motifs. These motifs corresponded to approximately 55.5% of the dinucleotide motifs. Among trinucleotide repeats, ATC (33.8%) was the most common motif, followed by AAC (17.9%), AGG (14.0%) and AAT (11.4%). The most abundant tetranucleotide motif was AAAC (22.8%), while AAAAT (15.0%) and AGCAGG (14.8%) were the most abundant repeat motifs for pentanucleotides and hexanucleotides, respectively.

**Table 3:** Summary of simple sequence repeat (SSR) types in the *P. yessoensis* transcriptome.

SSR Type	No. of SSR-containing ESTs	No. of SSRs	% of total SSRs
Di-nucleotides	557	580	21.1%
Tri-nucleotides	936	1,084	39.4%
Tetra-nucleotides	404	426	15.5%
Penta-nucleotides	387	401	14.6%
Hexa-nucleotides	245	257	9.4%
Total	2,480	2,748	100%

**Figure 3:**
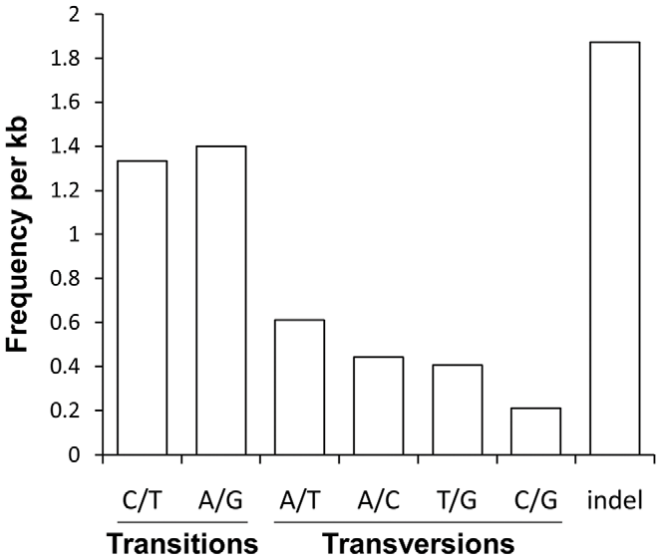
Classification of single nucleotide polymorphisms (SNPs) identified in the *P. yessoensis* transcriptome. The overall frequency of these SNP types in *P. yessoensis* transcriptome is one per 156 bp.

Potential SNPs were detected using the QualitySNP program. We identified 34,841 high-quality SNPs and 14,358 indels from 10,107 contigs (Fig. 3). The predicted SNPs included 20,958 transitions, 12,804 transversions. The overall frequency of all types of SNPs in the transcriptome, including indels, was one per 156 bp. Of the predicted SNPs, 40,063 (81.9%) were identified from contigs covered by ten or more reads , suggesting majority of SNPs identified in this study were covered at sufficient sequencing depth and more likely represent ‘true’ SNPs. Among the SNPs, 31,696 (64.4%) were identified from contigs with annotation information. These SNPs would also be priority candidates for maker development and should be very useful for further genetic or genomic studies on this species.

In conclusion, we first performed *de novo* transcriptome sequencing for the Yesso scallop *P. yessoensis* using the 454 GS FLX platform. A large number of candidate genes potentially involved in growth, reproduction, and stress/immunity-response were identified, and are worthy of further investigation. A large number of SNPs and SSRs were also identified and ready for marker development. This resource should lay an important foundation for future genetic or genomic studies on this species.

## Methods

### Scallop materials and RNA extraction

Adult individuals of *P. yessoensis* were obtained from Dalian Zhangzidao Fishery Group Corporation (Dalian Province, China) in 2009. Tissues including adductor muscle, digestive gland, male and female gonad, were dissected from adult scallops. To obtain larval materials, fertilization and larval cultures were performed according to [40]. Fertilized eggs were reared at 15°C. Larval samples were collected at different developmental stages, including blastula, gastrula, trochophore and D-shaped larva stages. All samples were flash frozen in liquid nitrogen and stored at −80°C until analysis. Total RNAs were extracted from these materials using the method described in [41]. The quantity and quality of total RNA was analyzed using an Ultrospec™ 2100 *pro* UV/Visible Spectrophotometer (Amersham Biosciences, Uppsala, Sweden) and gel electrophoresis. Equal quantities of high-quality RNA from each material were pooled for cDNA synthesis.

### cDNA library construction and 454 sequencing

cDNA samples were prepared following the protocol described in [21]. Briefly, first-strand cDNA was synthesized using SuperScript II reverse transcriptase (Invitrogen, CA, USA) with a modified oligo-dT primer (Cap-TRSA-CV) and a template-switch primer: SMART II™ A Oligonucleotide (Clontech, CA, USA). Then cDNA was amplified using PCR Advantage II polymerase (Clontech, CA, USA) and the following profile: 94°C for 5 minutes and 17 cycles of 94°C for 40 seconds, 65°C for 1 minute, and 72°C for 6 minutes. Multiple PCRs were performed for each library. The cDNA samples were then pooled and purified using the TIANquick Midi Purification kit (TIANGEN, Beijing, China).

Larval library was normalized using the Trimmer Direct kit (Evrogen, Moscow, Russia) to prevent over-representation of the most common transcripts. In contrast, tissue libraries were not normalized (for future comparison of transcript expression profiles among tissues). cDNA samples were sheared by sonication using a GA92-II D sonicator (Shangjia Biotechnology, Wuxi, China) to produce fragments of approximately 300–1,000 bp, which is appropriate fragment size range for 454 sequencing.

Oligonucleotide adaptors were ligated to the fragmented cDNA. One adaptor contained a barcode sequence that was used to discriminate samples from different libraries. Finally, all libraries were combined into a single pool. Approximately 5 µg of the mixed cDNA pool was used for high throughput sequencing using a 454 GS FLX sequencer (Roche, Basel, Switzerland).

### Sequence data analysis and assembly

The raw reads obtained were first pre-processed by removing the PolyA tails and adaptors using custom Perl scripts. All sequences smaller than 60 bases were eliminated based on the assumption that small reads might represent sequencing artifacts [21]. The trimmed and size-selected reads were then assembled using the publicly available program CAP3 [42], which can utilize quality scores to aid read assembly. The overlap settings used for this assembly were 40 bp and 80% similarity, with all other parameters set to their default values.

### Sequence annotation

The assembled sequences were compared against the NCBI non-redundant (Nr) protein database and Swiss-Prot database using BlastX with an E-value of 1e-4. Gene names were assigned to each assembled sequence based on the best BLAST hit (highest score). To increase computational speed, such search was limited to the first 10 significant hits for each query.

To annotate the assembled sequences with GO terms describing biological processes, molecular functions and cellular components, the Swiss-Prot BLAST results were imported into Blast2GO [43]–[45], a software package that retrieves GO terms, allowing gene functions to be determined and compared. These GO terms are assigned to query sequences, producing a broad overview of groups of genes cataloged in the transcriptome for each of three ontology vocabularies, biological processes, molecular functions and cellular components. The obtained annotation was enriched and refined using ANNEX [46], Validate Annotations and GO Slim [47], [48] integrated in the Blast2GO software. The data presented herein represent a GO analysis at level 2, illustrating general functional categories.

KEGG pathways were assigned to the assembled sequences using the online KEGG Automatic Annotation Server (KAAS), http://www.genome.jp/kegg/kaas/. The bi-directional best hit (BBH) method was used to obtain KEGG Orthology (KO) assignment [49]. The output of KEGG analysis includes KO assignments and KEGG pathways that are populated with the KO assignments.

### SSR and SNP discovery

SciRoko program v3.3 [50] was used to identify and localize microsatellite motifs. We searched for all types of SSRs from dinucleotides to hexanucleotides using default settings. Potential SNPs were detected using QualitySNP [51]. SNP identification was accomplished using a separate procedure from the main annotation pipeline. All the clean reads were first assembled using the CAP3 program, for which the overlap settings were 100 bp and a 95% similarity. SNP identification was limited to clusters containing at least four reads.

## Supporting Information

Table S1 **Sequences with significant BLAST matches against Nr and Swiss-Prot database.**
(XLS)

Table S2 **KEGG biochemical mappings for** ***P. yessoensis*** **.**
(DOC)

Table S3 **Candidate genes involved in growth, reproduction, stimulus response and immune defense.**
(XLS)
