# Supplementary material for: Transcriptome Sequencing and De Novo Analysis for Yesso Scallop (Patinopecten yessoensis) Using 454 GS FLX
Source: PLoS One. 2011 Jun 24;6(6):e21560. doi: 10.1371/journal.pone.0021560 (PMC3123371; doi:10.1371/journal.pone.0021560)
Supplement: Table S2 — KEGG biochemical mappings for P. yessoensis . (DOC) [file pone.0021560.s002.doc]

**Table S2. KEGG biochemical mappings for *P. yessoensis***

| KEGG categories represented | Unique sequences (Number of enzymes) |
| --- | --- |
| **Metabolism** | **2075 (1,308)** |
| Enzyme Families | 448 (320) |
| Carbohydrate Metabolism | 389 (223) |
| Amino Acid Metabolism | 389 (223) |
| Energy Metabolism | 266 (172) |
| Nucleotide Metabolism | 257 (134) |
| Metabolism of Cofactors and Vitamins | 243 (145) |
| Lipid Metabolism | 235 (134) |
| Glycan Biosynthesis and Metabolism | 189 (128) |
| Metabolism of Other Amino Acids | 124 (65) |
| Xenobiotics Biodegradation and Metabolism | 124 (61) |
| Biosynthesis of Secondary Metabolites | 87 (49) |
| Biosynthesis of Polyketides and Nonribosomal Peptides | 10 (4) |
| **Genetic Information Processing** | **2203 (1,448)** |
| Replication and Repair | 769 (483) |
| Folding, Sorting and Degradation | 634 (468) |
| Transcription | 524 (400) |
| Translation | 416 (200) |
| **Environmental Information Processing** | **739 (578)** |
| Signal Transduction | 324 (246) |
| Signaling Molecules and Interaction | 297 (248) |
| Membrane Transport | 172 (129) |
| **Cellular Processes** | **888 (626)** |
| Cell Motility | 227 (151) |
| Cell Growth and Death | 189 (125) |
| Immune System | 181 (133) |
| Endocrine System | 179 (126) |
| Transport and Catabolism | 175 (128) |
| Cell Communication | 155 (109) |
| Nervous System | 75 (58) |
| Circulatory System | 58 (47) |
| Development | 43 (32) |
| Sensory System | 8 (8) |
| Behavior | 8 (8) |
| **Total** | **4,846 (3,209)** |
